# How Can the Light Reactions of Photosynthesis be Improved in Plants?

**DOI:** 10.3389/fpls.2012.00199

**Published:** 2012-08-28

**Authors:** Dario Leister

**Affiliations:** ^1^Plant Molecular Biology, Department Biology I, Ludwig-Maximilians-University MunichPlanegg-Martinsried, Germany

*The evolutionary patchwork nature of the light reactions of photosynthesis in plants provides ample scope for their improvement, particularly with respect to its light-harvesting components and the susceptibility of photosystems to photodamage. Such improvements can be achieved by genetic engineering and, more indirectly, by conventional breeding, whereas synthetic biology should allow in the long-term the redesign or de novo creation of entire photosystems that are more efficient because they are less susceptible to photodamage and produce fewer harmful reactive oxygen species. This photosystem redesigning will require novel model organisms in which such concepts can be realized, tested, and reiteratively improved*.

## How “Perfect” is Photosynthesis?

The idea that plant photosynthesis cannot be improved, because evolution has already perfected it, is surprisingly widespread. I do not share this notion, for several reasons. (1) Natural selection, which maximizes total fitness rather than (agronomic) yield, has shaped plant photosynthesis for life in environments that differ considerably from the resource-rich settings provided by modern agriculture. (2) Even assuming that plant photosynthesis has been optimized during evolution, major parts of the original “hardware” were obviously sub-optimal, and have been bypassed rather than replaced. This is because plant photosynthesis originated in prokaryotes, and initially evolved in low-light (i.e., marine) conditions in the absence of oxygen. In consequence, plants have to cope with an evolutionary inheritance that includes a photosystem II (PSII), which is damaged by high concentrations of its substrate – light (Nishiyama et al., 2011; Murata et al., 2012). To avoid photodamage, plants have had to develop a whole set of regulatory and protective responses, including processes that dissipate excess excitation energy as heat and allow for rapid repair and turnover of photodamaged PSII subunits. These mechanisms are wasteful under natural conditions and possibly even unnecessary or disadvantageous under the artificial conditions employed in agriculture. Likewise, as an adaptation to the intense irradiation experienced by land plants, the light-harvesting complexes (LHCs) replaced the original cyanobacterial light-harvesting system (phycobilisomes), which was optimized to harvest low levels of light. Ironically, when land plants had to re-adapt to low-light conditions with the advent of woodland and forest canopy, they evolved the capacity to increase light-harvesting by appressing thylakoid membranes into grana stacks (Mullineaux, 2005), but whether grana are as efficient as the original phycobilisomes in harvesting low-light intensities is not at all clear. In the carbon fixation arm of photosynthesis, RuBisCO, the key enzyme of the Calvin-Benson Cycle, is a prominent example for another flawed photosynthetic component. RuBisCO is very inefficient and wastes lots of energy by using O_2_ (which was absent from the atmosphere when the enzyme was invented) as well as CO_2_ as a substrate.

Thus, the patchwork nature of photosynthesis provides, at least in theory, ample scope for improvement of the light reactions, particularly with respect to its light-harvesting components and the susceptibility of photosystems to photodamage.

## Potential Targets for Conventional Breeding

Conventional breeding requires intraspecific variation. Given the high degree of conservation of the structural components of the light reactions among land plants, the fact that there is almost no natural variation in basic photosynthetic parameters among different accessions of the model plant *Arabidopsis*, as determined by chlorophyll fluorescence parameters (El-Lithy et al., 2005), comes as no surprise. However, some natural variation has been found in mechanisms of constitutive protection against PSII photodamage (Jansen et al., 2010) and in non-photochemical quenching (NPQ; Jung and Niyogi, 2009). Increased vegetative biomass observed in hybrids between *Arabidopsis* accessions is only indirectly associated with an increase in photosynthesis, because the rate of photosynthesis per unit leaf area in parents and hybrids turns out to be constant (Fujimoto et al., 2012). Instead, heterosis is due to the larger cell size in hybrids, with correspondingly more chloroplasts and more chlorophyll per cell (Fujimoto et al., 2012).

It can therefore be concluded that the structural components of the light reactions of photosynthesis are very probably not susceptible to improvement by breeding, whereas mechanisms involved in the regulation or protection of the photosynthetic light reactions might be amenable to adjustment (Table 1).

**Table 1:** **Overview of approaches to improving photosynthetic light reactions**.

**Approaches and targets**	**Reference**
**BREEDING**	
NPQ	Jung and Niyogi (2009)
PSII photoinhibition	Jansen et al. (2010)
Unknown regulators of photosynthesis-associated traits	Fujimoto et al. (2012)
**GENETIC ENGINEERING**	
Modification/exchange of light-harvesting systems	Reviewed in: Blankenship et al. (2011)
Overexpression of endogenous or heterologous proteins	Chida et al. (2007), Pesaresi et al. (2009)
Inactivation of (wasteful) regulatory processes	Pribil et al. (2010)
**SYNTHETIC BIOLOGY**	
Redesign of photosensitive photosystem subunits	This study
Novel single-subunit photosystems without assembly	This study
Novel pigments introduced by synthetic amino acids	This study

Whereas breeding and genetic engineering exploit pre-existing intra- and interspecific variation, respectively, entirely novel amino acids, genes, proteins and pigments can be employed in synthetic biology approaches. By some definitions, the exchange of entire light-harvesting systems can be considered to lie within the ambit of synthetic biology.

## Potential Targets for Genetic Engineering

One obvious route to the improvement of carbon fixation is the transfer of genes from one species to another. Attempts to introduce C4 photosynthesis or other carbon-concentrating systems into C3 plants, engineer improved versions of RuBisCO, or even replace the entire Calvin-Benson cycle, are ongoing (reviewed in: Blankenship et al., 2011; Langdale, 2011). But how can the light reactions of photosynthesis in plants benefit from replacing components by their counterparts from other species? The most pronounced differences between the light reactions in photoautotrophic oxygen-evolving organisms reside in their light-harvesting antenna systems. For instance, cyanobacteria, glaucophytes, and red algae contain the aforementioned phycobilisomes, whereas land plants use LHCs. To enhance light-harvesting indirectly, research efforts are underway in crop plants to enable more light to penetrate to lower levels of the canopy. These involve either modifying plant architecture or decreasing chlorophyll content (reviewed in: Blankenship et al., 2011). The introduction of prokaryotic pigments that absorb further into the near-infrared (Blankenship et al., 2011), or even entire prokaryotic light-harvesting systems (to supplement or replace LHCs), into plants might make it possible to expand the absorption spectrum of photosynthesis and thus increase photosynthetic efficiency at low-light levels.

Moreover, the potential of genetic engineering is not restricted to the modification or replacement of LHCs. The removal or overexpression of single components of the photosynthetic light reactions can improve the efficiency of photosynthesis, at least under certain conditions. For instance, overexpression of either plastocyanin, the soluble electron transporter that reduces photosystem I (PSI), or its algal substitute cytochrome c_6_, increases biomass in *A. thaliana* (Chida et al., 2007; Pesaresi et al., 2009; Table 1). Similarly, increased phosphorylation of thylakoid proteins, achieved by inactivating the thylakoid phosphatase TAP38, improves photosynthetic electron flow under certain light conditions (Pribil et al., 2010). This indicates that simple single-gene genetic engineering of photosynthetic light reaction might have practical applications. Moreover, these findings, together with the observation that manipulation of photosynthetic carbon fixation in *Arabidopsis* (by introducing a prokaryotic glycolate catabolic pathway to bypass photorespiration) also increases biomass accumulation (Kebeish et al., 2007), argue strongly against the idea that plants are limited in their sink, but not in their source, capacity (reviewed in: Kant et al., 2012). Nevertheless, it is clear that neutralization of the feedback mechanisms that down-regulate photosynthesis when sink capacity becomes limiting, and increasing sink capacity *per se*, are both necessary to get the most out of improved photosynthetic light reactions.

Hence, genetic engineering of the light reactions of photosynthesis should focus primarily on modifying light-harvesting and regulators of photosynthetic electron flow, as well as on increasing the sink capacity of plants to cope with an enhanced photosynthetic rate. The resulting plants should exhibit more efficient photosynthesis under controlled conditions, e.g., in greenhouses, or in regions that cannot otherwise be extensively used for agriculture because of their short growing seasons.

## Potential Targets for Synthetic Biology

Whereas some of the opportunities for genetic engineering described above might be defined as synthetic biology approaches, a true synthetic biology project would aim to design photosystems that are insensitive to damage by light. This will need novel “hardware” and, therefore, will require the replacement of the light-sensitive subunits, or even the complete remodeling of photosystems, in particular PSII (Table 1). Assuming that wholesale remodeling is feasible, the problem arises of what to do when more excitation energy is available than is needed for carbon fixation? In natural photosynthesis, excess excitation energy is dissipated as heat, not only to prevent damage to the photosystems but to also to avoid the generation of reactive oxygen species (ROS). Therefore, a redesigned photosystem must not only be light-stable but also avoid ROS production. The latter attribute might be very difficult to realize, and require the design of additional “devices” that utilize or quench excess excitation energy. Hence, one may hazard the guess that several versions of photosystem cores and light-harvesting antenna will have to be designed and combined to fulfill the needs of different organisms and habitats. Such a redesigned photosystem core will probably not be a multiprotein-pigment complex like the natural photosystems. Instead, the number of proteins in such a redesigned photosystem might have to be dramatically reduced to avoid the complex assembly processes that operate in plants, which require a plethora of assembly factors – not all of which have been identified. Moreover, pigments might be introduced into such a novel photosystem by using synthetic amino acids with novel side-chains that absorb light. Finally, these novel photosystems might be used not only in the classical Z-scheme to produce ATP and NADPH, but also exploit novel ways to convert charge separation into chemical energy.

## Final Remarks

The considerations outlined above suggest that the light reactions of photosynthesis can be improved by genetic engineering and, more indirectly, by conventional breeding. The redesign or *de novo* creation of entire photosystems that are less susceptible to photodamage and produce fewer harmful ROS, is a formidable challenge. However, the gain in photosynthetic efficiency when photosystems require less repair and photoprotection will be significant. It is clear that crop plants are the least suited test systems for such approaches, given their long life cycle and inaccessibility to efficient genetic engineering technologies. Therefore, redesigning photosystems will not only require a very deep understanding of the structure and dynamics of the natural photosynthetic light reactions, but also open-minded and innovative concepts of what a perfect photosystem should look like, as well as novel model organisms in which such concepts can be realized, tested, and reiteratively improved.
